# Distinct Cross-Vascular Association Patterns of Carotid Plaque Burden and Vulnerability Features with Coronary Atherosclerotic Burden in Patients Undergoing Carotid Revascularization

**DOI:** 10.3390/jcm15207853

**Published:** 2026-10-12

**Authors:** Sunan Xu, Zhenjia Wang, Xinyu Wu, Lu Li, Wei Yu

**Affiliations:** Department of Radiology, Beijing Anzhen Hospital, Capital Medical University, No. 2 Anzhen Road, Chaoyang District, Beijing 100029, China; drxusunan@mail.ccmu.edu.cn (S.X.);

**Keywords:** carotid plaque, high-resolution magnetic resonance imaging, coronary atherosclerosis, carotid revascularization

## Abstract

**Background/Objectives**: This study aimed to investigate the associations between preoperative carotid high-resolution magnetic resonance imaging (HR-MRI) plaque features and coronary atherosclerotic burden, and to compare the cross-vascular association patterns of plaque burden-related measures and individual vulnerable plaque features. **Methods**: This retrospective study included consecutive patients who underwent carotid revascularization and had both preoperative carotid HR-MRI and coronary computed tomography angiography (CTA) between April 2017 and December 2024. Carotid plaque characteristics included calcification, lipid-rich necrotic core (LRNC), intraplaque hemorrhage (IPH), loose matrix (LM), ulceration, maximum wall thickness (MWT), maximum wall area (MWA), and normalized wall index (NWI). Coronary atherosclerotic burden was characterized using coronary artery calcium score (CACS), maximum luminal stenosis, and the number of diseased coronary arteries; the number of coronary arteries with ≥50% stenosis was additionally recorded. Associations between carotid plaque characteristics and coronary atherosclerotic burden were evaluated using correlation analyses and multivariable logistic regression. **Results**: A total of 162 patients were included (mean age, 66.11 ± 7.54 years; 138 [85.2%] men). CACS ≥ 400 was present in 76 patients (46.9%), obstructive coronary disease (≥50% stenosis in at least one coronary artery) in 96 patients (59.3%), and involvement of ≥3 diseased coronary arteries in 128 patients (79.0%). Greater coronary atherosclerotic burden was consistently associated with higher carotid calcification volume and MWT and NWI (all *p* < 0.05). After adjustment for clinical risk factors and correction for multiple testing, calcification volume, MWT and NWI remained associated with CACS ≥ 400, obstructive coronary disease, and involvement of ≥3 diseased coronary arteries. In contrast, individual vulnerable plaque features, including LRNC, IPH, LM, and ulceration, showed weaker and less consistent associations. **Conclusions**: In patients undergoing carotid revascularization, preoperative carotid HR-MRI-derived plaque burden-related features showed more consistent associations with coronary atherosclerotic burden than individual vulnerable plaque features. These findings suggest that plaque burden-related and vulnerable plaque features may reflect different aspects of atherosclerosis, with plaque burden-related measures more closely reflecting cumulative systemic atherosclerotic involvement and vulnerable plaque features primarily characterizing local plaque composition and instability.

## 1. Introduction

Cardiovascular disease (CVD) remains a leading cause of morbidity and mortality worldwide [1]. Atherosclerosis is a systemic disease that commonly affects multiple vascular territories, particularly the coronary and carotid arteries. The coexistence of coronary and carotid atherosclerosis is frequent and reflects an increased systemic atherosclerotic burden, highlighting the importance of cross-vascular assessment for comprehensive cardiovascular risk evaluation.

Coronary artery calcium score (CACS) is an established marker of coronary atherosclerotic burden and a strong predictor of coronary artery disease (CAD) [2,3,4]. Previous studies have demonstrated associations between coronary and carotid atherosclerosis, with CACS being correlated with carotid intima–media thickness, carotid plaque presence and plaque burden assessed by ultrasonography or computed tomography angiography (CTA) [5,6,7,8]. Contemporary vascular imaging modalities provide complementary perspectives on carotid atherosclerosis. Ultrasonography is widely used for evaluating luminal stenosis and plaque burden because of its accessibility and noninvasive nature, whereas CTA provides detailed assessment of vascular anatomy, calcification, plaque morphology, and luminal narrowing. High-resolution magnetic resonance imaging (HR-MRI) directly depicts the vessel wall with high soft-tissue contrast and permits detailed assessment of plaque morphology and tissue composition [9,10,11]. In the coronary circulation, coronary CTA enables noninvasive assessment of luminal stenosis, plaque burden and composition, thereby providing a broader characterization of coronary atherosclerosis than CACS alone [12,13].

In addition to vessel-wall morphology, HR-MRI can identify major plaque components, including intraplaque hemorrhage (IPH), lipid-rich necrotic core (LRNC), and loose matrix (LM), as well as morphological features such as ulceration, while also allowing quantitative measurements of vessel-wall and plaque burden [9,10,11]. Although vulnerable plaque components have been widely recognized as markers of local plaque instability, whether these individual features reflect systemic atherosclerotic involvement across different vascular territories remains less well established. In contrast, quantitative plaque burden-related measures may reflect cumulative atherosclerotic involvement rather than local plaque vulnerability alone. Whether these two categories of carotid imaging features show different cross-vascular associations with coronary atherosclerotic burden remains unclear.

Patients undergoing carotid revascularization have advanced carotid atherosclerosis and frequently have concomitant coronary artery disease, providing a clinically relevant setting in which to investigate cross-vascular imaging relationships. However, the extent to which carotid HR-MRI characteristics are associated with coronary atherosclerotic burden in this population remains insufficiently defined.

Therefore, this study aimed to investigate the associations between preoperative carotid HR-MRI-derived plaque characteristics and coronary atherosclerotic burden in patients undergoing carotid revascularization. Specifically, we compared the associations of quantitative plaque burden-related measures and individual vulnerable plaque features with multiple measures of coronary atherosclerotic burden. These analyses were intended to characterize differences in the cross-vascular associations of these carotid HR-MRI features.

## 2. Materials and Methods

### 2.1. Study Design and Population

This retrospective study was conducted in accordance with the Declaration of Helsinki [8], and approved by the institutional review board (Approval No. 2025265x). The requirement for written informed consent was waived.

**Figure 1:**
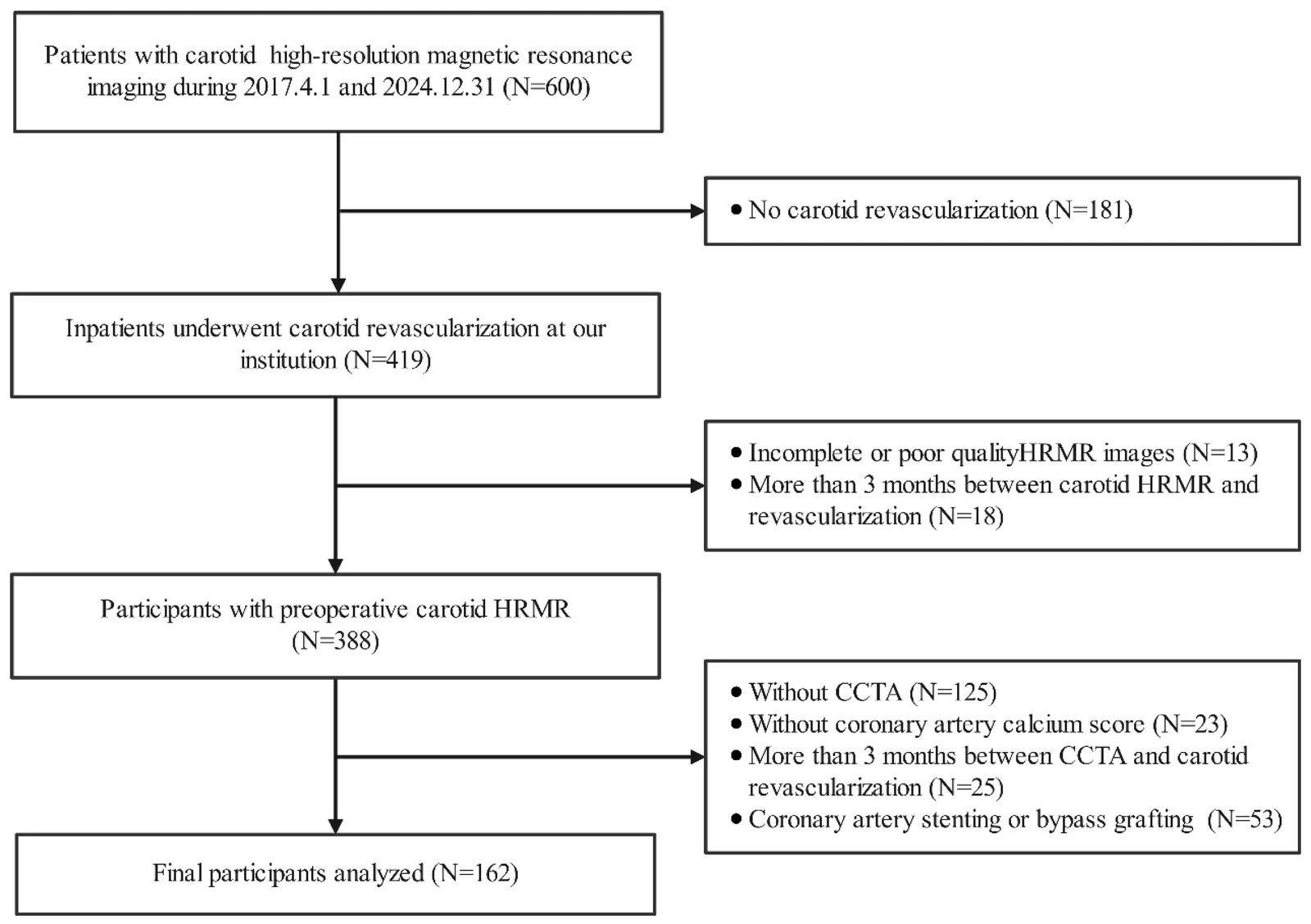
Flowchart of patient selection.

Consecutive hospitalized patients who underwent carotid revascularization between 1 April 2017 and 31 December 2024 were retrospectively screened. Patients with both preoperative carotid HR-MRI and coronary CTA were subsequently assessed for eligibility for the present cross-vascular analysis. Coronary CTA was obtained as part of clinical care when coronary assessment was considered appropriate during preoperative cardiovascular evaluation and was therefore not routinely performed in all patients. Patients were excluded based on the following criteria: (1) incomplete or poor-quality carotid HR-MRI; (2) an interval of more than 3 months between carotid HR-MRI and carotid revascularization; (3) unavailable coronary CTA or CACS data; (4) an interval of more than 3 months between coronary CTA and carotid revascularization; and (5) prior coronary revascularization, including coronary artery stenting or coronary artery bypass grafting. Detailed numbers and reasons for exclusion at each stage of patient selection are shown in Figure 1.

### 2.2. Clinical and Laboratory Data Collection

Demographic information, cardiovascular risk factors, and laboratory measurements were retrieved from electronic medical records. Smoking history was defined as current or former smoking, and alcohol use history was defined as current or former alcohol consumption. Hypertension was identified in patients with repeated systolic blood pressure ≥ 140 mmHg and/or diastolic blood pressure ≥ 90 mmHg, or in those receiving antihypertensive therapy. Diabetes mellitus was defined as fasting plasma glucose ≥ 7.0 mmol/L, random plasma glucose ≥ 11.1 mmol/L, or the use of glucose-lowering medication. Dyslipidemia was defined as total cholesterol ≥ 6.19 mmol/L, low-density lipoprotein cholesterol ≥ 4.14 mmol/L, triglycerides ≥ 2.27 mmol/L, high-density lipoprotein cholesterol < 1.04 mmol/L, or the use of lipid-lowering therapy. Body mass index (BMI) was calculated as weight in kilograms divided by height in meters squared. Medical records were also reviewed for a history of CVD, including myocardial infarction, angina pectoris, and heart failure, as well as prior cerebrovascular events.

### 2.3. HR-MRI Acquisition and Plaque Assessment

Carotid HR-MRI was performed on a 3.0-T scanner (Ingenia CX, Philips Healthcare, Best, The Netherlands) using a 32-channel head coil together with an 8-channel neck coil (TSImaging Healthcare, Beijing, China). Imaging covered both carotid bifurcations and was centered at the level of the largest carotid plaque. The standardized examination required approximately 30 min.

The protocol consisted of three-dimensional time-of-flight magnetic resonance angiography (3D TOF MRA), two-dimensional T1-weighted imaging (2D T1WI), two-dimensional T2-weighted imaging (2D T2WI), three-dimensional simultaneous non-contrast angiography and intraplaque hemorrhage (SNAP), and contrast-enhanced T1WI. A gadolinium-based contrast agent was administered intravenously at a dose of 0.1 mmol/kg and an injection rate of 3 mL/s, followed by a 20 mL saline flush. Detailed acquisition parameters are summarized in Table 1. Image quality was classified as excellent, good, marginal, and poor according to signal-to-noise ratio and imaging artifacts, and examinations classified as poor were excluded from further analysis [14].

**Figure 2:**
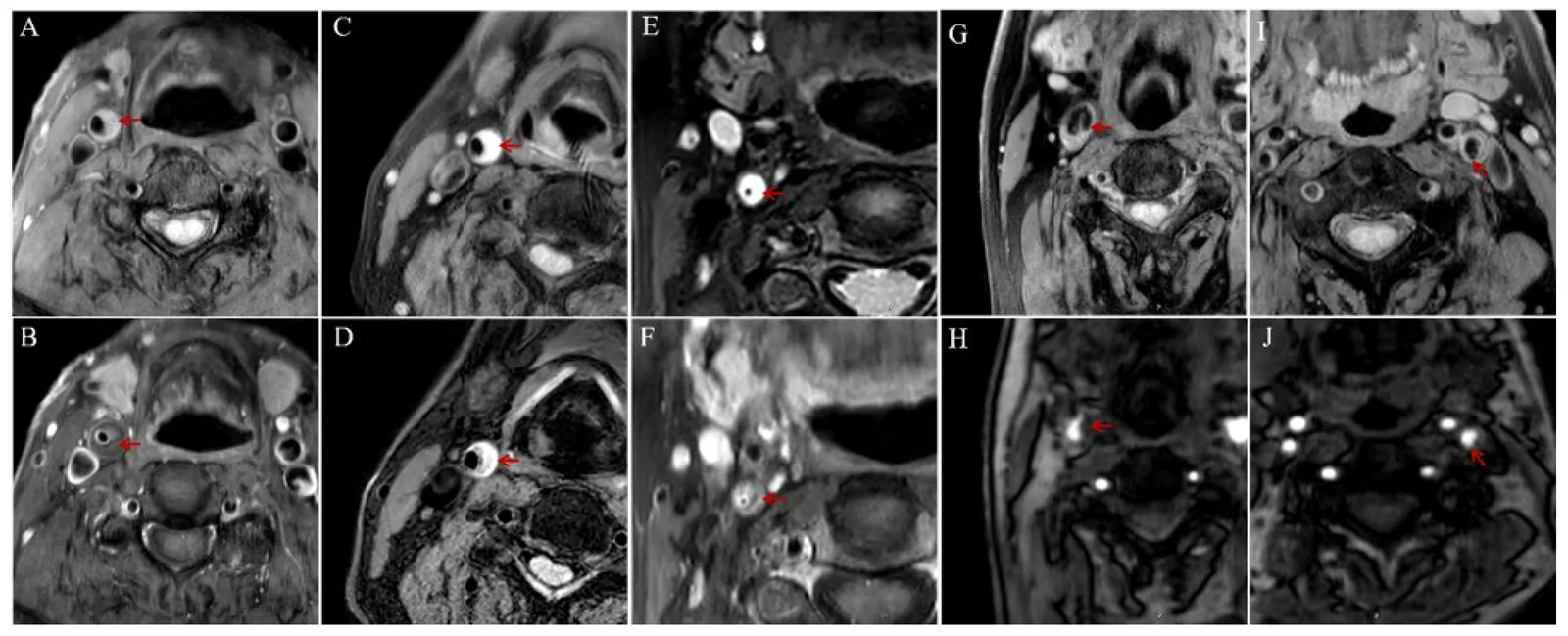
Representative HR-MRI images of the carotid plaque components. (**A**,**B**) LRNC on T1WI and contrast-enhanced T1WI. (**C**,**D**) IPH on T1WI and SNAP. (**E**,**F**) LM on T2WI and contrast-enhanced T1WI. (**G**,**H**) Calcification on T1WI and TOF. (**I**,**J**) Plaque ulceration on T1WI and TOF. Red arrows indicate the corresponding plaque components or morphological features. HR-MRI, high-resolution magnetic resonance imaging; LRNC, lipid-rich necrotic core; IPH, intraplaque hemorrhage; LM, loose matrix; T1WI, T1-weighted imaging T2WI, T2-weighted imaging; SNAP, simultaneous non-contrast angiography and intraplaque hemorrhage; TOF, time of flight.

Images analysis was performed using dedicated vessel wall software (Vessel Explorer 2.0, TSImaging Healthcare, Beijing, China). The lumen and outer vessel-wall contours were manually traced on pre-contrast black-blood T1WI and T2WI. Wall area was calculated as the difference between total vessel area and lumen area. The normalized wall index (NWI) was calculated as wall area divided by total vessel area and multiplied by 100 [NWI (%) = wall area/total vessel area × 100], where total vessel area was defined as the area enclosed by the outer vessel-wall boundary [15]. Plaque components were characterized according to their sequence-specific signal pattern. IPH was identified as a hyperintense region relative to the adjacent sternocleidomastoid muscle on SNAP images [16]. LRNC was defined as a plaque region showing absent or slight enhancement on contrast-enhanced T1WI relative to T1WI [17]. Calcification was identified as a hypointense region across all sequences [18]. Ulceration was defined as a luminal indentation into the plaque measuring >1.0 mm in width or >2.0 mm in depth and was confirmed on 3D TOF and contrast-enhanced T1WI, with hyperintense signal used to differentiate ulceration from juxtaluminal calcification [19]. LM was characterized as a hyperintense region on both T2WI and contrast-enhanced T1WI [20]. Representative HR-MRI images of these plaque components are shown in Figure 2. The volumes of IPH, LRNC, LM, and calcification were automatically derived from the manually delineated bilateral plaque regions and the corresponding slice thickness.

Two radiologists with more than 3 years of experience in vascular imaging (S.N. Xu and L. Li) independently evaluated the examinations without access to the patients’ clinical information. Final measurements were based on consensus, and discrepancies were resolved by a third neuroradiologist with more than 5 years of experience (Z.J. Wang). Interobserver reproducibility was assessed in 17 cases by an additional radiologist (T.T. Li). The intraclass correlation coefficients were 0.86 for calcification, 0.88 for LRNC, 0.80 for IPH, 0.92 for MWT, 0.90 for MWA, and 0.83 for NWI. The kappa coefficient for ulceration was 0.90.

### 2.4. Coronary CTA Acquisition and Analysis

Coronary CTA was acquired using either a Revolution CT scanner (GE Healthcare, Milwaukee, WI, USA) or a Somatom Definition Flash scanner (Siemens Healthcare, Forchheim, Germany) with prospective electrocardiogram-gating. Scan parameters included a gantry rotation time of 250–350 ms, tube voltage of 100–120 kV, and automated tube current modulation. Iopromide (50–60 mL, Ultravist, 370 mg I/mL; Bayer Schering Pharma, Berlin, Germany) was administered intravenously at 4–5 mL/s and followed by a 30–35 mL saline flush. Images were reconstructed at a slice thickness of 0.5–0.625 mm with a reconstruction increment of 0.5 mm.

Coronary calcium was quantified using the Agatston–Janowitz (AJ-130) method based on calcified lesions throughout the coronary artery tree [21]. According to commonly used CACS categories [22], patients were initially classified as having no calcification (0), mild calcification (1–99), moderate calcification (100–399), or severe calcification (≥400). Because relatively few patients had a CACS of 0, those with a score of 0 were combined with the 1–99 group for subsequent analyses.

Coronary atherosclerotic involvement was evaluated in the right coronary artery, left anterior descending artery, left circumflex artery, and left main coronary artery. A diseased coronary artery was defined as a major coronary artery with any atherosclerotic plaque on CTA, irrespective of the degree of luminal stenosis, and the number of diseased coronary arteries was recorded, with a maximum of four involved arteries. Thus, the number of disease coronary arteries reflected the extent of plaque-bearing coronary territories rather than the number of vessels with obstructive stenosis. Maximum coronary luminal stenosis was categorized as <50% or ≥50%, with ≥50% considered obstructive coronary disease [23]. The number of coronary arteries with stenosis ≥ 50% was also recorded. The study cohort was categorized according to CACS (0–99, 100–399, and ≥400), maximum coronary luminal stenosis (<50% and ≥50%), and the number of diseased coronary arteries (0–2, 3, and 4 arteries). These classifications were selected to capture complementary dimensions of coronary atherosclerosis: calcific plaque burden, luminal stenosis severity, and the extent of atherosclerotic involvement across the major coronary arteries.

### 2.5. Statistical Analysis

Normally distributed continuous variables are presented as mean ± standard deviation (SD), whereas variables with non-normal distributions are reported as median and interquartile range [IQR]. Categorical variables are expressed as counts and percentages. Distributional normality was evaluated using the Kolmogorov–Smirnov test. Two-group comparisons were performed using the Student’s *t* test or Mann–Whitney U test, as appropriate. Comparisons among multiple groups were performed using one-way analysis of variance (ANOVA) or Kruskal–Wallis H test, as appropriate. When the overall test was statistically significant, pairwise comparisons were performed using Tukey’s honestly significant difference test following ANOVA or Dunn’s test with Bonferroni adjustment following the Kruskal–Wallis test. Categorical variables were compared using the chi-square test or Fisher exact test, as appropriate. To assess potential selection associated with coronary CTA availability, baseline characteristics were additionally compared between patients included in the final analysis and those without coronary CTA.

Cross-vascular associations between carotid plaque characteristics and coronary atherosclerotic burden were examined using Pearson or Spearman correlation coefficients, as appropriate. Separate multivariable logistic regression models were fitted for each carotid plaque characteristic. The three primary coronary outcomes were CACS ≥ 400, maximum coronary luminal stenosis ≥ 50%, and involvement of ≥3 diseased coronary arteries. All models were adjusted for age, sex, hypertension, diabetes mellitus, dyslipidemia, and history of CVD. To limit model complexity, only one carotid plaque characteristic was included in each multivariable model together with the prespecified clinical covariates; therefore, MWT, MWA, and NWI were not entered simultaneously into the same model. Continuous imaging variables were standardized before regression analysis, and ORs were reported per 1-SD increase, whereas plaque ulceration was modeled as a binary variable (present versus absent). To address multiplicity, the Benjamini–Hochberg false discovery rate (FDR) procedure was applied across the primary tests comprising eight carotid plaque characteristics and three coronary outcomes, and both nominal *p*-value and FDR-adjusted q-value were reported. To assess the potential influence of outcome dichotomization, complementary sensitivity analyses were performed using ordinal logistic regression. CACS was modeled using the ordered categories of 0–99, 100–399, and ≥400; maximum coronary luminal stenosis was modeled according to its ordered severity categories; and coronary disease extent was modeled using 0–2, 3, and 4 diseased coronary arteries. The same modeling strategy and covariate adjustment as in the primary analyses were applied. Potential nonlinearity on the logit scale was assessed by adding a quadratic term for each continuous imaging variable to the corresponding model. Multicollinearity was assessed using variance inflation factors (VIFs) for the predictors included in the models. Complete regression coefficients (β), adjusted ORs with 95% CIs, nominal *p*-values, and FDR-adjusted q-values for the primary analyses are provided in Supplementary Table S3, whereas results of the ordinal sensitivity analyses are provided in Supplementary Table S4.

All statistical analyses were performed using IBM SPSS Statistics (version 26.0; IBM Corp., Chicago, IL, USA). All tests were two-sided, and a *p* value < 0.05 was considered statistically significant.

## 3. Results

### 3.1. Study Population

A total of 162 patients were included, including 138 (85.2%) men, with a mean age of 66.11 ± 7.54 years. According to CACS, 48 patients were classified as 0–99, 38 as 100–399, and 76 as ≥400. Maximum coronary luminal stenosis was <50% in 66 patients and ≥50% in 96 patients. Regarding the extent of plaque-bearing coronary territories, 34 patients had 0–2 diseased coronary arteries, 54 had 3 diseased arteries, and 74 had 4 diseased arteries. Baseline characteristics across these classification schemes are presented in Supplementary Table S1A–C. Baseline characteristics of the included cohort and patients without available coronary CTA are compared in Supplementary Table S2. Carotid plaque ulceration was present in 70 (43.2%) patients. Compared with women, men demonstrated a higher prevalence of smoking and alcohol use and larger LRNC and IPH volumes and MWA (all *p* < 0.05; Table 2), whereas no significant sex differences were observed for the remaining clinical and imaging characteristics.

### 3.2. Association Between Coronary Atherosclerotic Burden and Carotid Plaque Characteristics

Cross-vascular associations were examined by comparing carotid plaque characteristics across categories of CACS, maximum coronary luminal stenosis, and the number of diseased coronary arteries. Overall, plaque burden-related parameters showed a more consistent pattern of association across these coronary measures than individual vulnerable plaque features.

Compared with patients with CACS 0–99, those with CACS 100–399 or ≥400 had greater calcification volume and higher MWT, MWA, and NWI after multiplicity-adjusted pairwise comparisons (all *p* < 0.05; Table 3). MWT and NWI were higher in patients with CACS ≥ 400 than in those with CACS 100–399 (Table 3). In contrast, no significant differences were observed in individual vulnerable plaque features, including LRNC, IPH, LM volume, or plaque ulceration across CACS categories (*p* > 0.05).

Similarly, patients with maximum coronary luminal stenosis ≥ 50% showed greater carotid calcification volume (*p* = 0.007) and higher MWT and NWI values than those with stenosis <50% (both *p* < 0.05, Table 4). Moreover, patients with 4 diseased coronary arteries involvement exhibited greater carotid calcification volume and higher MWT and NWI values than those with 0–2 diseased coronary arteries, while patients with 3 diseased coronary arteries also showed higher MWT and NWI values than those with 0–2 diseased coronary arteries (all *p* < 0.05; Table 5). Conversely, vulnerable plaque features, including LRNC, IPH, LM, and ulceration, were not significantly associated with coronary disease extent.

**Figure 3:**
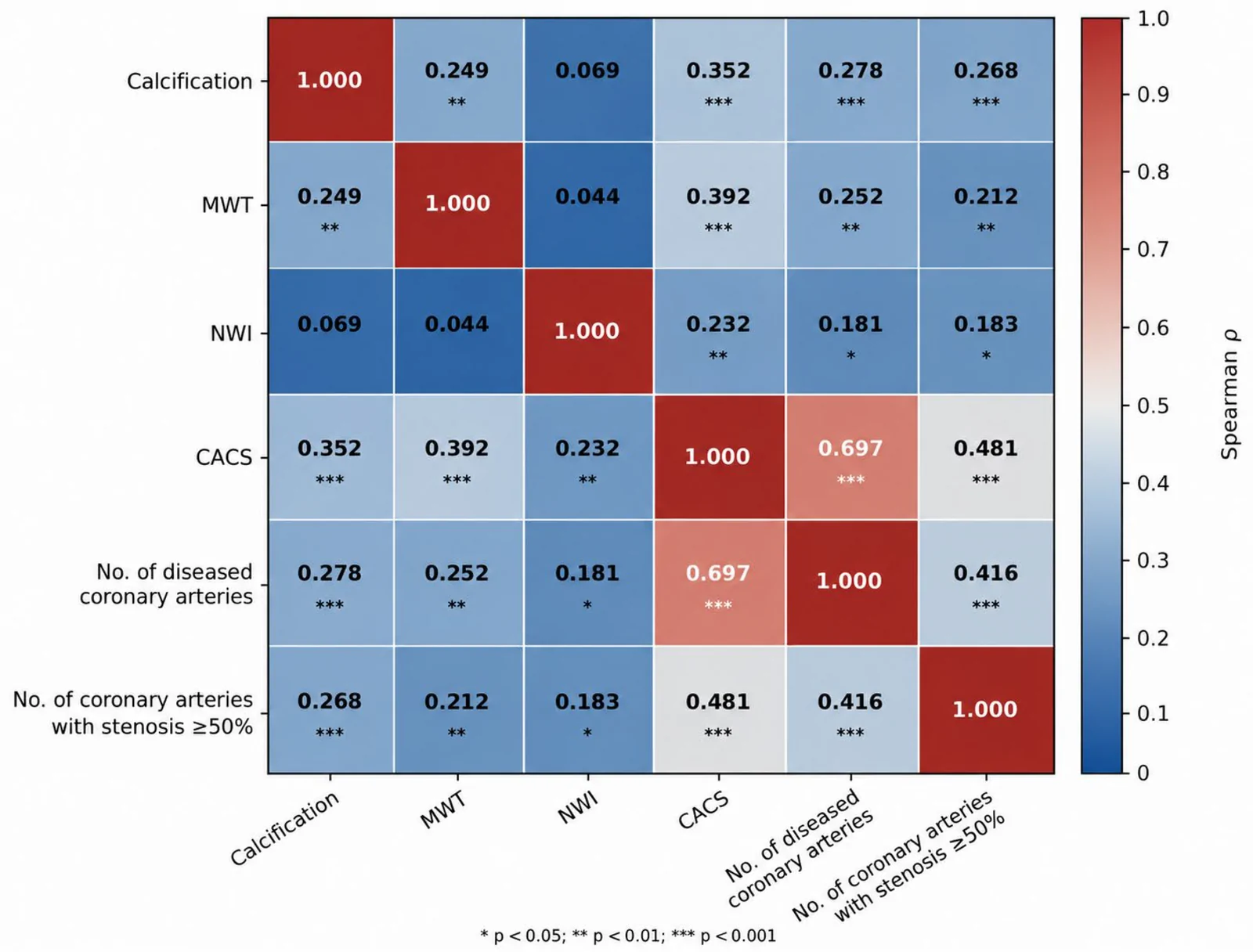
Correlation matrix illustrating the cross-vascular associations between carotid plaque burden-related features and coronary atherosclerotic burden. The matrix includes carotid calcification volume, MWT, NWI, CACS, the number of diseased coronary arteries, and the number of coronary arteries with ≥50% stenosis. Values within the matrix represent correlation coefficients; * *p* < 0.05, ** *p* < 0.01, and ***p < 0.001. MWT, maximum wall thickness; NWI, normalized wall index; CACS, coronary artery calcium score.

Significant correlations were observed between the principal carotid plaque burden measures and coronary atherosclerotic burden. Specifically, carotid calcification volume, MWT, and NWI were significantly correlated with CACS, the number of diseased coronary arteries, and the number of coronary arteries with stenosis ≥ 50% (all *p* < 0.05; Figure 3).

**Figure 4:**
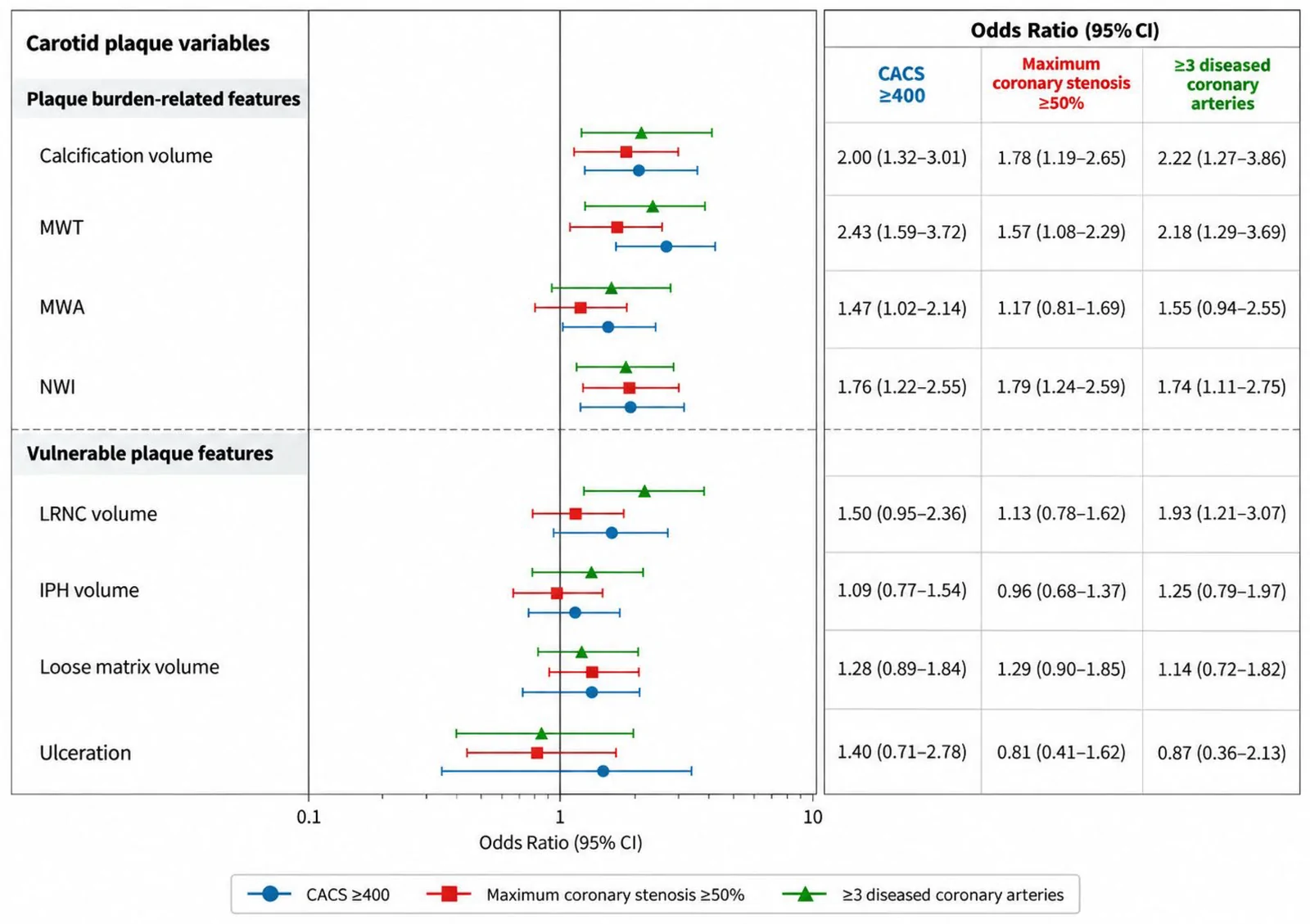
Adjusted associations of carotid plaque features with coronary atherosclerotic burden. Forest plots show odds ratios (ORs) and 95% confidence intervals (CIs) for calcification volume, MWT, MWA, and NWI, LRNC volume, IPH volume, LM volume, and ulceration in relation to CACS ≥ 400, maximum coronary luminal stenosis ≥ 50%, and involvement of ≥3 diseased coronary arteries. Each carotid plaque characteristics was evaluated in a separate multivariable logistic regression model. Continuous imaging variables are presented per 1-SD increase, whereas ulceration is modeled as a binary variable. Models were adjusted for age, sex, hypertension, diabetes mellitus, dyslipidemia, and history of CVD. Points represent adjusted ORs, horizontal lines indicate 95% CIs, and the vertical line represents an OR of 1. CACS, coronary artery calcium score; MWT, maximum wall thickness; MWA, maximum wall area; NWI, normalized wall index; LRNC, lipid-rich necrotic core; IPH, intraplaque hemorrhage; LM, loose matrix.

To determine whether these associations were independent of clinical risk factors, multivariable logistic regression analyses were performed after adjustment for age, sex, hypertension, diabetes, dyslipidemia, and CVD history. Each carotid plaque characteristic was evaluated in a separate model. After Benjamini–Hochberg FDR correction, plaque burden-related characteristics, particularly calcification volume, MWT, and NWI, remained consistently associated with greater coronary atherosclerotic burden across the three primary coronary outcomes. Among individual vulnerable plaque features, LRNC volume remained significantly associated with involvement of ≥3 diseased coronary arteries, whereas IPH, LM, and ulceration showed no significant adjusted associations. (Figure 4 and Supplementary Table S3). No significant evidence of nonlinearity was identified for the continuous imaging predictors, and no multicollinearity was observed in the multivariable models. Sensitivity analyses preserving the ordered information of coronary disease severity showed a broadly consistent overall pattern, with calcification volume, MWT, and NWI remaining the most consistently associated plaque burden-related characteristics across alternative outcome specifications. Detailed results are presented in Supplementary Table S4.

## 4. Discussion

To our knowledge, this is the first study to comprehensively evaluate the associations between carotid HR-MRI-derived plaque characteristics and coronary atherosclerotic burden in patients undergoing carotid revascularization. We found that carotid plaque burden-related features, particularly calcification volume, MWT and NWI, were consistently associated with multiple indicators of coronary atherosclerotic burden, including CACS, obstructive coronary artery disease, and involvement of ≥3 diseased coronary arteries. In contrast, individual vulnerable plaque features, including LRNC, IPH, LM, and ulceration, showed weaker and less consistent associations. Rather than indicating that plaque burden-related features are generally more important than vulnerable plaque features, these findings suggest that the two types of imaging features may reflect different aspects of atherosclerosis: plaque burden-related measures may more closely reflect cumulative systemic atherosclerotic involvement, whereas vulnerable plaque features mainly characterize local plaque composition and instability.

Atherosclerosis is increasingly recognized as a systemic inflammatory disease involving multiple vascular territories [24]. Previous studies investigating carotid–coronary associations have mainly relied on carotid ultrasound or CTA. Carotid ultrasonography is a well-established and reproducible modality for quantifying carotid atherosclerotic burden, including plaque thickness, area, and volume. Whereas HR-MRI provides more detailed characterization of plaque tissue composition and vessel-wall morphology [10]. Luminal stenosis alone may underestimate disease extent because of compensatory positive remodeling [12]. Recent studies have further explored the cross-vascular value of carotid HR-MRI. Qin et al. demonstrated that HR-MRI-derived Carotid Plaque-RADS was independently associated with coronary functional stenosis in patients undergoing carotid revascularization [25]. Our study extends these findings by quantitatively evaluating plaque burden-related measures and examining their associations with multiple dimensions of coronary atherosclerotic burden.

The observed association patterns may reflect the distinct biological information captured by these imaging markers. Quantitative plaque burden parameters, such as MWT and NWI, represent the cumulative consequences of atherosclerotic progression, including lipid accumulation, vascular remodeling, extracellular matrix alteration, and arterial wall expansion. Previous MRI studies have shown that quantitative carotid plaque measurements are associated with cardiovascular risk [26], and carotid plaque burden has also been associated with coronary calcification [27]. Carotid calcification may be particularly relevant in this context because vascular calcification reflects long-term arterial injury and remodeling involving osteogenic differentiation of vascular smooth muscle cells, inflammation, oxidative stress, and extracellular matrix changes [28]. These cumulative processes may help explain the consistent associations of calcification, MWT and NWI with coronary atherosclerotic burden in our cohort. The biological plausibility of these cross-vascular associations is further supported by shared pathogenic mechanisms between carotid and coronary atherosclerosis, including endothelial dysfunction, lipid retention, chronic inflammation, and vascular remodeling [24,29]. Longitudinal MRI studies have also shown that carotid plaque composition evolves over time, with calcified and multicomponent plaques representing different stages of plaque progression [30].

In contrast, vulnerable plaque features, including IPH, LRNC, LM, and ulceration, mainly reflect local plaque composition and instability. IPH is associated with intraplaque neovascularization, fragile microvessels, and inflammatory activation, whereas LRNC represents advanced lipid accumulation and necrotic transformation [29]. These features are important markers of plaque rupture and cerebrovascular events, but their distribution may vary substantially among lesions and vascular territories. Therefore, their less consistent associations with coronary atherosclerotic burden should not be interpreted as indicating lower clinical importance, but rather as reflecting the different information captured by local plaque vulnerability and cumulative plaque burden. From a practical perspective, ultrasonography may be sufficient for routine quantification of carotid plaque burden, whereas HR-MRI may provide additional information when simultaneous assessment of plaque burden and plaque composition is required. Whether ultrasound-derived plaque burden measures provide cross-vascular information comparable to HR-MRI-derived measures remains to be determined in direct comparative studies.

From a clinical perspective, patients undergoing carotid revascularization represent a high-risk population with advanced atherosclerosis and a high prevalence of concomitant coronary disease. Contemporary cardiovascular prevention increasingly emphasizes the complementary role of multimodality imaging in identifying subclinical cardiovascular disease and refining risk assessment beyond conventional clinical risk factors [31]. In this context, our findings suggest that HR-MRI-derived carotid plaque burden markers, particularly MWT, NWI, and calcification volume, may provide additional information regarding concomitant coronary atherosclerotic involvement and contribute to a more integrated cardiovascular and cerebrovascular assessment. Rather than replacing coronary CTA, carotid plaque phenotyping may serve as a complementary approach for characterizing systemic atherosclerotic involvement, and its clinical value requires further prospective validation.

Several limitations should be acknowledged. First, this was a single-center retrospective study with a relatively limited sample size, which may restrict statistical power and generalizability. In addition, the requirement for available coronary CTA may have introduced selection bias, as several clinical and carotid imaging characteristics differed between included patients and those without available coronary CTA. Therefore, the generalizability of our findings to the broader population undergoing carotid revascularization should be interpreted with caution, and external validation in larger multicenter cohorts is warranted. Second, the cross-sectional design precludes causal inference, and longitudinal studies are needed to determine whether carotid HR-MRI-derived plaque characteristics are associated with subsequent progression of coronary atherosclerosis or future cardiovascular events. Third, although CACS is an established marker of coronary atherosclerotic burden, it mainly reflects calcified plaque and does not fully capture non-calcified coronary plaque components; therefore, the relationship between carotid plaque features and total coronary plaque burden may be underestimated. Finally, although carotid HR-MRI plaque characterization has demonstrated good agreement with histopathology [18,32], potential misclassification of plaque components cannot be completely excluded.

## 5. Conclusions

In conclusion, carotid HR-MRI-derived plaque burden-related features, particularly calcification volume, MWT, and NWI, were associated with coronary atherosclerotic burden, whereas individual vulnerable plaque components showed less consistent cross-vascular associations. These findings support the distinction between cumulative systemic atherosclerotic burden and local plaque vulnerability, rather than suggesting that one type of plaque feature is more important than the other. In patients undergoing carotid revascularization who have already undergone carotid HR-MRI, greater carotid plaque burden may provide complementary information regarding concomitant coronary atherosclerotic involvement and may support a more integrated cardiovascular assessment. These imaging findings should be considered complementary to, rather than a substitute for, dedicated coronary imaging, and their clinical value requires further prospective validation.

## Figures and Tables

**Table 1:** Acquisition parameters of the carotid HR-MRI protocol.

Parameters	3D TOF	2D T1WI	2D T2WI	3D SNAP	Contrast-Enhanced T1WI
TE, ms	4	10	50	6.8	10
TR, ms	20	800	4800	12	800
FOV, mm × mm	160 × 160	160 × 160	160 × 160	160 × 160	160 × 160
Resolution, mm × mm	0.6 × 0.6	0.6 × 0.6	0.6 × 0.6	0.6 × 0.6	0.6 × 0.6
Slice thickness, mm	2	2	2	2	2
Number of slices	40	20	20	40	20

Note: 3D, three-dimensional; TOF, time of flight; 2D, two-dimensional; T1WI, T1-weighted imaging; T2WI, T2-weighted imaging; SNAP, simultaneous non-contrast angiography and intraplaque hemorrhage; TE, echo time; TR, repetition time; FOV, field of view.

**Table 2:** Baseline clinical and imaging characteristics of the study population.

Characteristics	All Subjects (N = 162)	Male (N = 138)	Female (N = 24)	*p*
Age, years	66.11 ± 7.54	66.01 ± 7.58	66.71 ± 7.40	0.675
BMI, kg/m^2^	25.16 ± 2.89	25.20 ± 2.59	24.79 ± 4.34	0.668
Smoking history (%)	91 (56.2)	88 (63.8)	3 (12.5)	<0.001
Alcohol use history (%)	57 (35.2)	55 (39.9)	2 (8.3)	0.003
Hypertension (%)	121 (74.7)	102 (73.9)	19 (79.2)	0.585
Diabetes (%)	59 (36.4)	50 (36.2)	9 (37.5)	0.925
Dyslipidemia (%)	95 (58.6)	82 (59.4)	13 (54.2)	0.680
History of CVD (%)	58 (35.8)	49 (35.5)	9 (37.5)	0.851
TC, mmol/L	3.80 (3.27, 4.50)	3.80 (3.22, 4.46)	3.91 (3.70, 5.51)	0.100
HDL-c, mmol/L	1.09 (0.92, 1.27)	1.07 (0.90, 1.24)	1.27 (1.02, 1.32)	0.082
LDL-c, mmol/L	2.06 (1.61, 2.60)	2.01 (1.60, 2.49)	2.30 (1.86, 2.83)	0.116
TG, mmol/L	1.25 (0.96, 1.61)	1.25 (0.91, 1.61)	1.29 (1.06, 1.64)	0.528
Coronary artery:				
CACS	410.30 (94.12, 851.92)	410.30 (87.67, 911.17)	160.93 (94.12, 760.97)	0.873
Stenosis: (%)				
<50%	66 (40.7)	58 (42.0)	8 (33.3)	0.424
≥50%	96 (59.3)	80 (58.0)	16 (66.7)	
Number of diseased coronary arteries (%)				
0–2	34 (21.0)	28 (20.3)	6 (25.0)	0.854
3	54 (33.3)	46 (33.3)	8 (33.3)	
4	74 (45.7)	64 (46.4)	10 (41.7)	
Carotid artery:				
Calcification (mm^3^)	62.84 (6.64, 159.46)	60.45 (5.81, 153.75)	59.63 (8.54, 165.43)	0.596
LRNC (mm^3^)	592.50 (316.15, 946.21)	611.10 (341.23, 955.47)	314.70 (200.20, 524.06)	<0.001
IPH (mm^3^)	170.32 (0.00, 478.54)	204.42 (0.00, 518.48)	0.00 (0.00, 269.66)	0.034
LM (mm^3^)	148.26 (65.24, 253.78)	148.26 (62.58, 249.60)	158.00 (51.24, 284.90)	0.777
Ulceration (%)	70 (43.2)	63 (45.7)	7 (29.2)	0.132
MWT (mm)	4.40 ± 0.79	4.43 ± 0.81	4.16 ± 0.75	0.126
MWA (mm^2^)	83.78 ± 27.38	85.74 ± 27.50	69.55 ± 24.81	0.008
NWI, %	87.50 (74.99, 94.84)	87.65 (75.19, 94.33)	83.86 (74.32, 95.87)	0.875

Data are presented as mean ± SD, median (IQR), or n (%), as appropriate. CVD, cardiovascular disease. TC, total cholesterol. HDL-c, high-density lipoprotein cholesterol. LDL-c, low-density lipoprotein cholesterol. TG, triglyceride. CACS, coronary artery calcium score. LRNC, lipid-rich necrotic core. IPH, intraplaque hemorrhage. LM, loose matrix. MWT, maximum wall thickness. MWA, maximum wall area. NWI, normalized wall index.

**Table 3:** Carotid plaque characteristics according to CACS categories.

Characteristics	CACS 0–99 (N = 48)	CACS 100–399 (N = 38)	CACS ≥ 400 (N = 76)	*p*
Calcification (mm^3^)	1.34 (0.00, 48.97)	76.38 (16.50, 168.18) **	88.65 (22.17, 182.27) ***	<0.001
LRNC (mm^3^)	546.69 (302.75, 865.39)	574.48 (266.47, 790.85)	709.30 (353.54, 1069.00)	0.077
IPH (mm^3^)	91.11 (0.00, 454.00)	218.65 (0.00, 472.52)	269.28 (0.00, 619.48)	0.229
LM (mm^3^)	155.85 (44.73, 262.94)	122.13 (43.80, 243.04)	146.16 (68.37, 254.83)	0.661
Ulceration (%)	16 (33.3)	17 (44.7)	37 (48.7)	0.238
MWT (mm)	3.97 ± 0.70	4.32 ± 0.87 *	4.70 ± 0.70 *** †	<0.001
MWA (mm^2^)	72.69 ± 29.17	87.11 ± 27.57 *	88.18 ± 25.09 **	0.006
NWI, %	82.21 (72.15, 89.08)	87.60 (73.26, 93.03) ***	89.83 (83.18, 96.81) *** †	<0.001

Data are presented as mean ± SD, median (IQR), or n (%), as appropriate. Pairwise comparisons: * *p* < 0.05, ** *p* < 0.01, and *** *p* < 0.001 versus the lowest CACS group; † *p* < 0.05 versus the intermediate CACS group. Pairwise comparisons were performed using Tukey’s honestly significant difference test following ANOVA or Dunn’s test with Bonferroni adjustment following the Kruskal–Wallis test, as appropriate. CACS, coronary artery calcium score; LRNC, lipid-rich necrotic core; IPH, intraplaque hemorrhage; LM, loose matrix; MWT, maximum wall thickness; MWA, maximum wall area; NWI, normalized wall index.

**Table 4:** Carotid plaque characteristics according to maximum coronary luminal stenosis.

Characteristics	<50% Stenosis (N = 66)	≥50% Stenosis (N = 96)	*p*
Calcification (mm^3^)	26.60 (0.00, 92.32)	69.45 (11.43, 179.13)	0.007
LRNC (mm^3^)	623.53 (338.26, 887.02)	596.76 (317.03, 951.71)	0.814
IPH (mm^3^)	192.69 (0.00, 475.89)	224.03 (0.00, 527.30)	0.876
LM (mm^3^)	100.40 (40.22, 257.22)	164.63 (86.76, 251.15)	0.060
Ulceration (%)	31 (47.0)	39 (40.6)	0.423
MWT (mm)	4.19 ± 0.86	4.54 ± 0.73	0.006
MWA (mm^2^)	80.77 ± 30.77	85.11 ± 25.30	0.328
NWI, %	84.02 (72.44, 93.45)	88.51 (80.62, 95.92)	0.003

Data are presented as mean ± SD, median (IQR), or n (%), as appropriate. LRNC, lipid-rich necrotic core; IPH, intraplaque hemorrhage; LM, loose matrix; MWT, maximum wall thickness; MWA, maximum wall area; NWI, normalized wall index.

**Table 5:** Carotid plaque characteristics according to the number of diseased coronary arteries.

Characteristics	0–2 Arteries (N = 34)	3 Arteries (N = 54)	4 Arteries (N = 74)	*p*
Calcification (mm^3^)	12.61 (0.00, 53.35)	54.99 (0.00, 125.59)	88.65 (28.35, 183.84) ***	<0.001
LRNC (mm^3^)	608.29 (179.40, 873.30)	620.78 (341.05, 919.08)	587.67 (347.44, 955.87)	0.336
IPH (mm^3^)	0.00 (0.00, 411.97)	260.60 (0.00, 595.33)	249.57 (0.00, 498.00)	0.146
LM (mm^3^)	168.92 (54.93, 257.79)	155.13 (49.57, 265.83)	128.40 (61.71, 227.65)	0.670
Ulceration (%)	13 (38.2)	23 (42.6)	34 (45.9)	0.749
MWT (mm)	3.94 ± 0.82	4.42 ± 0.80 *	4.58 ± 0.72 **	<0.001
MWA (mm^2^)	75.29 ± 32.91	87.10 ± 25.68	84.29 ± 26.01	0.138
NWI, %	78.17 (70.30, 88.74)	87.65 (79.60, 95.05) *	88.84 (76.98, 95.60) *	0.009

Data are presented as mean ± SD, median (IQR), or n (%), as appropriate. Pairwise comparisons: * *p* < 0.05, ** *p* < 0.01, and *** *p* < 0.001 versus the 0–2 diseased coronary artery group. Pairwise comparisons were performed using Tukey’s honestly significant difference test after ANOVA or Dunn’s test with Bonferroni adjustment after the Kruskal–Wallis test, as appropriate. LRNC, lipid-rich necrotic core; IPH, intraplaque hemorrhage; LM, loose matrix; MWT, maximum wall thickness; MWA, maximum wall area; NWI, normalized wall index.

## Data Availability

The datasets generated and analyzed during the current study are not publicly available due to institutional regulations and patient privacy restrictions but may be available from the corresponding authors upon reasonable request and with appropriate institutional approval.

## Supplementary Materials

The following supporting information can be downloaded at: https://www.mdpi.com/article/10.3390/jcm15207853/s1, Table S1A: Baseline characteristics according to CACS categories; Table S1B: Baseline characteristics according to maximum coronary luminal stenosis; Table S1C: Baseline characteristics according to number of diseased coronary arteries; Table S2: Comparison of baseline characteristics between patients included in the final analysis and patients without available coronary CTA; Table S3: Multivariable logistic regression analyses of carotid plaque characteristics in relation to coronary atherosclerotic burden; Table S4: Sensitivity analyses of the associations between carotid plaque characteristics and ordinal measures of coronary atherosclerotic burden.

## Abbreviations

The following abbreviations are used in this manuscript:

CEA	Carotid endarterectomy
CAS	Carotid artery stenting
CAD	Coronary artery disease
CACS	Coronary artery calcium score
CVD	Cardiovascular disease
CTA	Computed tomography angiography
HR-MRI	High-resolution magnetic resonance imaging
IPH	Intraplaque hemorrhage
LRNC	Lipid-rich necrotic core
LM	Loose matrix
MWA	Maximum wall area
MWT	Maximum wall thickness
NWI	Normalized wall index
