# Supplementary material for: Distinct Cross-Vascular Association Patterns of Carotid Plaque Burden and Vulnerability Features with Coronary Atherosclerotic Burden in Patients Undergoing Carotid Revascularization
Source: J Clin Med. 2026 Oct 12;15(20):7853. doi: 10.3390/jcm15207853 (PMC13705445; doi:10.3390/jcm15207853)
Supplement: Supplementary file 1 [file jcm-15-07853-s001.zip › jcm-4554790-supplementary.pdf]

Supplementary Table S1A. Baseline characteristics according to CACS categories.

Characteristics	CACS 0-99 (N=48)	CACS 100-399 (N=38)	CACS ≥400 (N=76)	*p*
Age, years	63.50 ± 7.89	67.66 ± 7.43	66.99 ± 7.02	0.014
Male sex, n (%)	40 (83.3)	32 (84.2)	66 (86.8)	0.850
BMI, kg/m^2	25.04 ± 3.10	25.47 ± 3.29	25.01 ± 2.66	0.742
Smoking history, n (%)	30 (62.5)	20 (52.6)	41 (53.9)	0.569
Alcohol use history, n (%)	19 (39.6)	10 (26.3)	28 (36.8)	0.405
Hypertension, n (%)	36 (75.0)	28 (73.7)	57 (75.0)	0.987
Diabetes, n (%)	9 (18.8)	17 (44.7)	33 (43.4)	0.009
Dyslipidemia, n (%)	24 (50)	23 (60.5)	48 (63.2)	0.379
History of CVD, n (%)	12 (25.0)	13 (34.2)	33 (43.4)	0.111
TC, mmol/L	3.80 (3.25, 4.69)	3.68 (3.17, 3.98)	3.97 (3.39, 4.47)	0.213
HDL-c, mmol/L	1.18 (0.94, 1.31)	1.11 (0.94, 1.23)	1.08 (0.92, 1.24)	0.512
LDL-c, mmol/L	1.95 (1.52, 2.69)	1.81 (1.55, 2.29)	2.20 (1.69, 2.63)	0.107
TG, mmol/L	1.39 (0.99, 1.85)	1.04 (0.86, 1.48)	1.29 (1.06, 1.59)	0.143

Supplementary Table S1B. Baseline characteristics according to maximum coronary luminal stenosis.

Characteristics	< 50% stenosis (N=66)	≥ 50% stenosis (N=96)	*p*
Age, years	65.03 ± 7.26	66.85 ± 7.67	0.131
Male sex, n (%)	58 (87.9)	80 (83.3)	0.424
BMI, kg/m^2	25.12 ± 2.77	25.14 ± 3.06	0.955
Smoking history, n (%)	37 (56.1)	54 (56.2)	0.981
Alcohol use history, n (%)	26 (39.4)	31 (32.3)	0.352
Hypertension, n (%)	49 (74.2)	72 (75.0)	0.913
Diabetes, n (%)	20 (30.3)	39 (40.6)	0.164
Dyslipidemia, n (%)	38 (57.6)	57 (59.4)	0.532
History of CVD, n (%)	22 (33.3)	36 (37.5)	0.706
TC, mmol/L	3.80 (3.31, 4.30)	3.85 (3.24, 4.48)	0.881
HDL-c, mmol/L	1.17 (1.03, 1.27)	1.05 (0.89, 1.23)	0.025
LDL-c, mmol/L	2.03 (1.57, 2.46)	2.12 (1.61, 2.61)	0.541
TG, mmol/L	1.21 (0.93, 1.54)	1.25 (0.97, 1.61)	0.598

Supplementary Table S1C. Baseline characteristics according to number of diseased coronary arteries.

Characteristics	0-2 arteries (N=34)	3 arteries (N=54)	4 arteries (N=74)	*p*
Age, years	62.85 ± 7.10	66.67 ± 8.47	67.20 ± 6.64	0.016
Male sex, n (%)	28 (82.4)	46 (85.2)	64 (86.5)	0.854
BMI, kg/m^2	24.45 ± 2.99	25.63 ± 3.21	25.08 ± 2.66	0.232
Smoking history, n (%)	22 (64.7)	26 (48.1)	43 (58.1)	0.282
Alcohol use history, n (%)	12 (35.3)	19 (35.2)	26 (35.1)	1.000
Hypertension, n (%)	26 (76.5)	41 (75.9)	54 (73.0)	0.898
Diabetes, n (%)	9 (26.5)	18 (33.3)	32 (43.2)	0.183
Dyslipidemia, n (%)	15 (44.1)	32 (59.3)	48 (64.9)	0.276
History of CVD, n (%)	7 (20.6)	17 (31.5)	34 (45.9)	0.028

TC, mmol/L	3.91 (3.27, 4.61)	3.69 (3.33, 4.11)	3.88 (3.01, 4.48)	0.969
HDL-c, mmol/L	1.22 (0.95, 1.36)	1.13 (0.94, 1.23)	1.05 (0.91, 1.25)	0.242
LDL-c, mmol/L	1.99 (1.39, 2.58)	1.95 (1.67, 2.45)	2.20 (1.63, 2.70)	0.544
TG, mmol/L	1.28 (0.97, 1.54)	1.25 (1.00, 2.10)	1.25 (0.90, 1.53)	0.528

Data are presented as mean ± SD, median (IQR), or n (%), as appropriate. BMI, body mass index; CACS, coronary artery calcium score; CVD, cardiovascular disease; TC, total cholesterol; HDL-c, high-density lipoprotein cholesterol; LDL-c, low-density lipoprotein cholesterol; TG, triglycerides.

Supplementary Table S2. Comparison of baseline characteristics between patients included in the final analysis and patients without available coronary CTA

Characteristics	Included cohort (N=162)	Excluded cohort without available coronary CTA (N=226)	*p*
Age, years	66.11 ± 7.54	65.24 ± 8.12	0.280
Male sex, n (%)	138 (85.2)	185 (81.9)	0.424
BMI, kg/m^2	25.16±2.89	25.15 ± 3.24	0.981
Smoking history, n (%)	91 (56.2)	159 (70.4)	0.006
Alcohol use history, n (%)	57 (35.2)	94 (41.6)	0.227
Hypertension, n (%)	121 (74.7)	154 (68.1)	0.183
Diabetes, n (%)	59 (36.4)	93 (41.2)	0.364
Dyslipidemia, n (%)	95 (58.6)	161 (71.2)	0.013
History of CVD, n (%)	58 (35.8)	118 (52.2)	0.002
TC, mmol/L	3.80 (3.27, 4.50)	3.64 (3.14–4.31)	0.271
HDL-c, mmol/L	1.09 (0.92, 1.27)	1.02 (0.87–1.19)	0.043
LDL-c, mmol/L	2.06 (1.61, 2.60)	1.98 (1.58–2.49)	0.410
TG, mmol/L	1.25 (0.96, 1.61)	1.22 (0.91–1.73)	0.405
Calcification volume, mm^3	62.84 (6.64, 159.46)	47.21 (0.00–80.34)	0.037
LRNC volume, mm^3	592.50 (316.15, 946.21)	359.98 (168.95–641.01)	0.021
IPH volume, mm^3	170.32 (0.00, 478.54)	0.00 (0.00–163.48)	0.001
LM volume, mm^3	148.26 (65.24, 253.78)	1.40 (0.00–87.99)	<0.001
Ulceration	70 (43.2)	90 (39.8)	0.507
MWT (mm)	4.40±0.79	4.33±0.85	0.419
MWA (mm^2)	83.78±27.38	78.49±25.21	0.053
NWI, %	87.50 (74.99, 94.84)	86.83 (73.81, 92.20)	0.054

Data are presented as mean ± SD, median (IQR), or n (%), as appropriate. BMI, body mass index; CVD, cardiovascular disease; TC, total cholesterol; HDL-c, high-density lipoprotein cholesterol; LDL-c, low-density lipoprotein cholesterol; TG, triglycerides; LRNC, lipid-rich necrotic core; IPH, intraplaque hemorrhage; LM, loose matrix.

Supplementary Table S3. Multivariable logistic regression analyses of carotid plaque characteristics in relation to coronary atherosclerotic burden

Carotid plaque characteristics	CACS ≥400				Maximum coronary luminal stenosis ≥50%				≥3 diseased coronary arteries			
	β	Adjusted OR (95% CI)	*p*	FDR *q*	*β*	Adjusted OR (95% CI)	*p*	FDR *q*	*β*	Adjusted OR (95% CI)	*p*	FDR *q*
Calcification volume	0.691	2.00 (1.32–3.01)	<0.001	0.011	0.575	1.78 (1.19–2.65)	0.005	0.017	0.796	2.22 (1.27–3.86)	0.005	0.017
MWT	0.889	2.43 (1.59–3.72)	<0.001	0.001	0.451	1.57 (1.08–2.29)	0.019	0.045	0.779	2.18 (1.29–3.69)	0.004	0.017
MWA	0.388	1.47 (1.02–2.14)	0.041	0.089	0.161	1.17 (0.81–1.69)	0.390	0.520	0.439	1.55 (0.94–2.55)	0.083	0.153
NWI, %	0.566	1.76 (1.22–2.55)	0.003	0.016	0.584	1.79 (1.24–2.59)	0.002	0.015	0.556	1.74 (1.11–2.75)	0.017	0.035
LRNC volume	0.406	1.50 (0.95–2.36)	0.079	0.153	0.120	1.13 (0.78–1.62)	0.519	0.651	0.658	1.93 (1.21–3.07)	0.006	0.017
IPH volume	0.082	1.09 (0.77–1.54)	0.643	0.701	−0.036	0.96 (0.68–1.37)	0.839	0.839	0.223	1.25 (0.79–1.97)	0.337	0.476
LM volume	0.249	1.28 (0.89–1.84)	0.179	0.287	0.255	1.29 (0.90–1.85)	0.169	0.287	0.134	1.14 (0.72–1.82)	0.570	0.651
Ulceration	0.339	1.40 (0.71–2.78)	0.332	0.476	−0.211	0.81 (0.41–1.62)	0.550	0.651	−0.135	0.87 (0.36–2.13)	0.766	0.799

Results are presented as regression coefficients (β), adjusted odds ratios (ORs) with 95% confidence intervals (CIs), nominal p-values, and false discovery rate (FDR)-adjusted q-values. Each carotid plaque characteristic was evaluated in a separate multivariable logistic regression model adjusted for age, sex, hypertension, diabetes mellitus, dyslipidemia, and history of cardiovascular disease. Continuous imaging variables were standardized before regression analysis; therefore, ORs are expressed per 1-SD increase. Plaque ulceration was modeled as a binary variable (present versus absent). The Benjamini–Hochberg FDR procedure was applied across the primary tests comprising eight carotid plaque characteristics and three coronary outcomes. LRNC, lipid-rich necrotic core; IPH, intraplaque hemorrhage; LM, loose matrix; MWT, maximum wall thickness; MWA, maximum wall area; NWI, normalized wall index; CACS, coronary artery calcium score; OR, odds ratio; CI, confidence interval; FDR, false discovery rate.

Supplementary Table S4. Sensitivity analyses of the associations between carotid plaque characteristics and ordinal measures of coronary atherosclerotic burden.

Carotid plaque characteristics	CACS category (0-99/100-399/≥400)			Maximum coronary luminal <50%/ ≥50%			Diseased coronary arteries (0-2/3/4)		
	Adjusted OR (95% CI)	*p*	FDR *q*	Adjusted OR (95% CI)	*p*	FDR *q*	Adjusted OR (95% CI)	*p*	FDR *q*
Calcification volume	2.08 (1.42–3.04)	<0.001	0.002	1.56 (1.11–2.18)	0.010	0.031	1.73 (1.21–2.47)	0.003	0.012
MWT	2.23 (1.55–3.20)	<0.001	<0.001	1.80 (1.29–2.52)	<0.001	0.004	1.55 (1.11–2.17)	0.010	0.031
MWA	1.60 (1.13–2.27)	0.009	0.031	1.28 (0.93–1.76)	0.138	0.254	1.10 (0.79–1.54)	0.555	0.620
NWI, %	1.81 (1.28–2.54)	0.001	0.004	1.48 (1.09–2.02)	0.013	0.034	1.44 (1.11–1.86)	0.011	0.047
LRNC volume	1.53 (1.06–2.20)	0.024	0.053	1.24 (0.92–1.68)	0.156	0.267	1.54 (1.07–2.22)	0.021	0.050
IPH volume	1.10 (0.80–1.52)	0.557	0.620	1.10 (0.81–1.48)	0.548	0.620	1.12 (0.81–1.54)	0.504	0.620
LM volume	1.21 (0.87–1.69)	0.262	0.393	1.10 (0.80–1.50)	0.568	0.620	1.12 (0.80–1.57)	0.502	0.620
Ulceration	1.51 (0.79–2.88)	0.208	0.332	0.85 (0.46–1.57)	0.615	0.641	1.09 (0.57–2.08)	0.793	0.793

Results are presented as adjusted odds ratios (ORs) with 95% confidence intervals (CIs), nominal p-values, and false discovery rate (FDR)-adjusted q-values. Each carotid plaque characteristic was evaluated in a separate regression model adjusted for age, sex, hypertension, diabetes mellitus, dyslipidemia, and history of cardiovascular disease. CACS was modeled using the ordered categories of 0–99, 100–399, and ≥400, and coronary disease extent was modeled using 0–2, 3, and 4 diseased coronary arteries. Maximum coronary luminal stenosis was analyzed as <50% versus ≥50%. Continuous imaging variables were standardized before regression analysis, and ORs are expressed per 1-SD increase; plaque ulceration was modeled as a binary variable (present versus absent). The Benjamini–Hochberg FDR procedure was applied across the sensitivity-analysis tests comprising eight carotid plaque characteristics and three coronary outcomes. For the ordinal outcomes, an OR >1 indicates higher odds of being in a more severe coronary atherosclerotic burden category. CACS, coronary artery calcium score; LRNC, lipid-rich necrotic core; IPH, intraplaque hemorrhage; LM, loose matrix; MWT, maximum wall thickness; MWA, maximum wall area; NWI, normalized wall index; OR, odds ratio; CI, confidence interval; FDR, false discovery rate.
